# Multisystem inflammatory syndrome in children associated with SARS-CoV-2: extracardiac radiological findings

**DOI:** 10.1259/bjr.20210570

**Published:** 2021-12-08

**Authors:** Berna Ucan, Seda Kaynak Sahap, Hasibe Gokce Cinar, Yasemin Tasci Yildiz, Cigdem Uner, Meltem Polat, Betul Emine Derinkuyu

**Affiliations:** 1 Department of Pediatric Radiology, Dr. Sami Ulus Gynecology, Obstetricsand Child Health and Diseases Training and Research Hospital, Ankara, Turkey; 2 Department of Pediatric Infectious Disease, Dr. Sami Ulus Gynecology, Obstetrics and Child Health and Diseases Training and Research Hospital, Ankara, Turkey

## Abstract

**Objective:**

Multisystem inflammatory syndrome in children (MIS-C) is seen as a serious delayed complication of severe acute respiratory syndrome coronavirus 2 (SARS-CoV-2) infection. The aim of this study was to describe the most common imaging features of MIS-C associated with SARS-CoV-2.

**Methods:**

A retrospective review was made of the medical records and radiological imaging studies of 47 children (26 male, 21 female) in the age range of 25 months–15 years who were diagnosed with MIS-C between August 2020 and March 2021. Chest radiographs were available for all 47 patients, thorax ultrasound for 6, chest CT for 4, abdominal ultrasound for 42, abdomen CT for 9, neck ultrasound for 4, neck CT for 2, brain CT for 1, and brain MRI for 3.

**Results:**

The most common finding on chest radiographs was perihilar–peribronchial thickening (46%). The most common findings on abdominal ultrasonography were mesenteric inflammation (42%), and hepatosplenomegaly (38%, 28%). Lymphadenopathy was determined in four patients who underwent neck ultrasound, one of whom had deep neck infection on CT. One patient had restricted diffusion and T2 hyperintensity involving the corpus callosum splenium on brain MRI, and one patient had epididymitis related with MIS-C.

**Conclusion:**

Pulmonary manifestations are uncommon in MIS-C. In the abdominal imaging, mesenteric inflammation, hepatosplenomegaly, periportal edema, ascites and bowel wall thickening are the most common findings.

**Advances in knowledge:**

The imaging findings of MIS-C are non-specific and can mimic many other pathologies. Radiologists should be aware that these findings may indicate the correct diagnosis of MIS-C.

## Introduction

Multisystem inflammatory syndrome in children (MIS-C) is a recently defined hyperinflammatory syndrome resulting from an immune-mediated delayed host response to the severe acute respiratory syndrome coronavirus 2 (SARS-CoV-2) infection. The clinical presentation of MIS-C consists of fever, abdominal symptoms, headaches, and mucocutaneous lesions such as rash and conjunctivitis, and may swiftly progress to shock and end organ injury.^1^

The imaging findings of MIS-C are non-specific and not well known as yet. Although this disease is a serious condition, it can be controlled, and organ damage can be prevented by the timely initiation of treatments.^2^

Radiologists and clinicians need to be familiar with the radiological imaging features and differential diagnoses of this disease to be able to provide a timely diagnosis and initiate appropriate treatment.

Several studies have recently reported clinical, laboratory and imaging findings of MIS-C associated with SARS-CoV-2.^3,4^ The aim of this study was to determine the most common imaging features of MIS-C associated with SARS-CoV-2.

## Methods and materials

This retrospective study was conducted in a regional pediatric hospital. The study was conducted in accordance with the Helsinki Declaration. Approval for the study was granted by the Ethics Review Board of Dr Sami Ulus Maternity and Paediatric Training and Research Hospital (E-21/04–148).

### Patients

The study included children aged <18 years who were diagnosed with MIS-C associated with SARS-CoV-2 in Dr. Sami Ulus Maternity and Children's Health and Diseases Training and Research Hospital between August 2020 and March 2021. The diagnosis of MIS-C was made in the inpatient clinical services according to the ‘Centers for Disease Control and Prevention’ (CDC) case definition criteria.^1^ Demographic information (age and gender) of the patients, laboratory findings (reverse transcribed SARS-CoV-2 PCR and serology), clinical findings (SARS-CoV-2 close contact history and symptoms during admission) were noted from the hospital database named Nucleus Medical Information system. Patients with a positive reverse transcribed SARS-CoV-2 test but did not meet the diagnostic criteria for MIS-C were not included in the study.

Organ/system involvement was described according to the clinical symptoms. Mucocutaneous findings were assessed based on skin rash and conjunctivitis. Respiratory system findings were cough and shortness of breath. Gastrointestinal system (GIT) findings were diarrhea, vomiting, and abdominal pain. Findings related to head and neck involvement were described as neck swelling and sore throat. Neurological system findings were lethargy, confusion, irritability, encephalopathy and seizures. Hematological system findings were neutrophilia, lymphopenia and/or thrombocytopenia. Any other additional clinical findings were also recorded. Since fever is a symptom which involves all systems, it was not included in any system.

### Image analysis

All imaging studies were initially independently reported during patient admission by pediatric radiologists with 5–15 years of experience and interpretations were made.

For every patient, every X-ray, Ultrasonography, CT and MRI image was reviewed from the Picture Archiving and Communication System (PACS) of our hospital by two fellowship trained pediatric radiologists blinded to the patient’s clinical data and the initial interpretation. The interpretations were made by consensus. Echocardiographic imaging was performed in all patients but was not reviewed for this study.

All chest radiographs taken at the time of admission and during hospitalization were reviewed. Findings on the radiographs with symptoms at the most severe point of the patient’s illness were recorded. On chest radiography, peribronchial thickening, perihilar or diffuse infiltration, consolidation, atelectasis, cardiomegaly, and pleural effusion were evaluated.

Chest CT (Siemens Healthineers, Erlangen Germany) was performed with intravenous contrast material. The presence of ground glass opacities, consolidation, and its distribution (unilateral or bilateral/ peripheral or diffuse), mosaic pattern, reverse halo sign, pulmonary embolism, hilar lymphadenopathy, atelectasis, and pleural effusion were evaluated.

Pleural effusion, atelectasis and consolidation were followed by thorax ultrasound. If these findings were seen on abdominal ultrasound, they were also noted.

Abdominal studies were assessed in four groups. First, size abnormalities of the liver, spleen, kidneys were noted using age-based normal sizes.^5^ Second, parenchymal signal abnormalities of the solid organs, which were defined as increased echogenicity of the liver and kidney, were recorded on ultrasound. Third, abnormalities of the hollow visceral organs, including wall thickening (accepted as >3 mm) of the gallbladder, bowel, appendix, and urinary bladder were noted. Fourth, periportal edema, mesenteric lymph nodes with signs of mesenteric inflammation and the presence of ascites were noted.

Neck ultrasound and CT images were evaluated for the presence of lymphadenopathy, deep neck infection, myositis, and salivary gland inflammation. Cranial CT and MRI performed on a patient with neurological symptoms included cerebellar ataxia, altered mental status and gait disturbance. Additional imaging studies were performed according to clinical symptoms, such as scrotal swelling in one patient.

### Statistical analysis

Normally distributed continuous variables such as age and gender, were expressed as median, SD, and range values. Imaging findings were summarized descriptively using absolute counts and percentages based on the final consensus interpretations. Statistical analysis was performed with Microsoft Excel.

## Results

Evaluation was made of 47 children who were diagnosed with MIS-C associated with SARS-CoV-2 in our hospital between August 2020 and March 2021. The patients comprised 26 (55%) males and 21 (45%) females with a median age of 8.6 years (range, 25 months–15 years).

**Figure 1.**
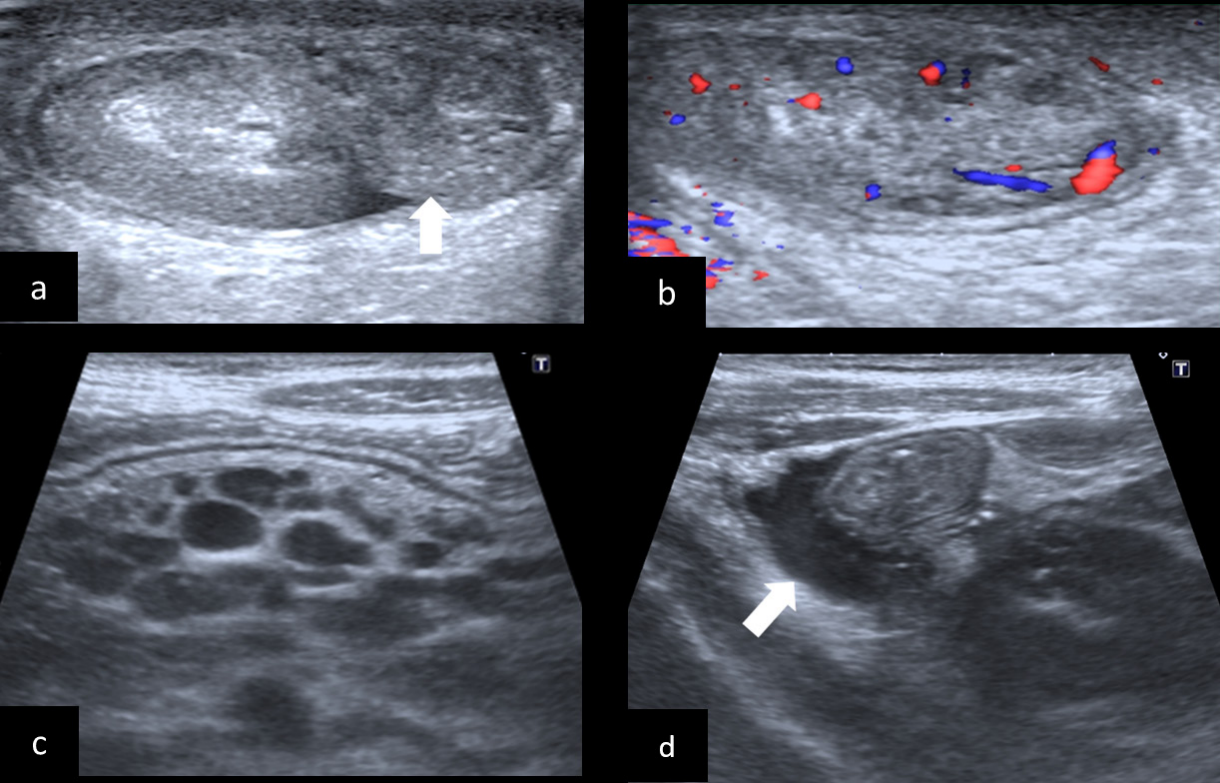
Imaging findings of an 8‐year‐old boy who presented with fever, scrotal pain and swelling. (a) Sagittal plane color Doppler and (b) Grayscale ultrasonography images demonstrate enlargement (white arrow) and hypervascularity of the epididymis consistent with epididymitis. (c) Abdominal ultrasonography of the same patient shows multiple mesenteric lymph nodes with mesenteric inflammation in the right lower quadrant and (d). small-volume ascites (white arrow).

The presenting clinical characteristics of patients are shown in Table 1. All patients (47/47, 100%) presented with fever. Other presenting signs and symptoms according to organ-system involvement included mucocutaneous in 43 (91%), GIT in 34 (72%), hematological in 33 (70%), head and neck in 21 (44%), respiratory in 12 (25%), and neurological in 3 (6%). Only one patient presented with complaints of scrotal pain and swelling in addition to abdominal pain (Figure 1). In our study, 33 (70%) of the children with MIS-C were older than 7 years old, and 14 (30%) were younger than 7 years old. Unlike older children, the most common presenting symptoms in young children, apart from fever, was related to the mucocutaneous and gastrointestinal system.

**Table 1.** Presenting clinical characteristics of patients admitted With MIS-C

Characteristic	Number (%)
Sex	
Male	26 (55)
Female	21 (45)
Age (y), mean	8.6
Presenting symptoms	
Fever	47
Mucocutaneous findings	43 (91)
GIS findings	34 (72)
Hematologic system findings	33 (70)
Head and neck findings	21 (44)
Respiratory system findings	12 (25)
Neurologic findings	3 (6)
Genitourinary system findings	1 (2)

MIS-C, multisystem inflammatory syndrome in children.

### Laboratory findings

The serological findings for SARS-CoV-2 of all the patients were positive in 45 patients (95%). Positive reverse transcription–polymerase chain reaction (RT-PCR) results and serological findings for SARS-CoV-2 were determined in 4 (8.5%) patients. There was no laboratory evidence of SARS-CoV-2 infection with negative RT-PCR and antibody results in 2 (4.2%) patients. These two patients had a history of contact with a COVID-19 case within the 4 weeks prior to the onset of symptoms. Contact history with a confirmed COVID-19 case within a median of 4 weeks before the onset of the symptoms was determined in 35 (74%) patients.

### Imaging findings

#### Abdominal imaging findings

The summary of the abdominal imaging findings for each modality are presented in Table 2.

**Table 2.** Abdominal imaging findings with MIS-C

Abdominal Imaging	Number (%)
Abdominal ultrasound	42
*Solid viscera abnormalities*	
Hepatomegaly	16 (38)
Splenomegaly	12 (28)
Hepatosplenomegaly	12 (28)
Periportal edema	11 (26)
Increased echogenicity of the liver	7 (16)
Echogenic kidneys	6 (14)
*Hollow viscera abnormalities*	
Ileocecal bowel wall thickening	9 (21)
Gallbladder wall thickening	3 (7)
*Peritoneal abnormalities*	
Mesenteric inflammation	18 (42)
Ascites	10 (24)
Mesenteric lymph nodes	7 (16)
*Chest abnormalities(incidental*)	
Pleural effusion	10 (24)
Lower atelectasis	1 (2)
CT, abdomen and pelvis	9
*Solid viscera abnormalities*	
Hepatomegaly	4 (44)
Splenomegaly	3 (33)
Periportal edema	2 (22)
Enlargement of kidneys	1 (11)
*Hollow viscera abnormalities*	
Urinary bladder wall thickening	4 (44)
İleocecal wall thickening	3 (33)
Gallbladder wall thickening	3 (33)
Colon wall thickening	1 (11)
Thickening of the appendix	1 (11)
*Peritoneal abnormalities*	
Mesenteric inflammation (increased density)	9 (100)
Ascites	6 (66)
Mesenteric lymph nodes	6 (66)
*Chest abnormalities(incidental*)	
Pleural effusion	3 (33)
Lower atelectasis	2 (22)

MIS-C, multisystem inflammatory syndrome in children.

**Figure 2.**
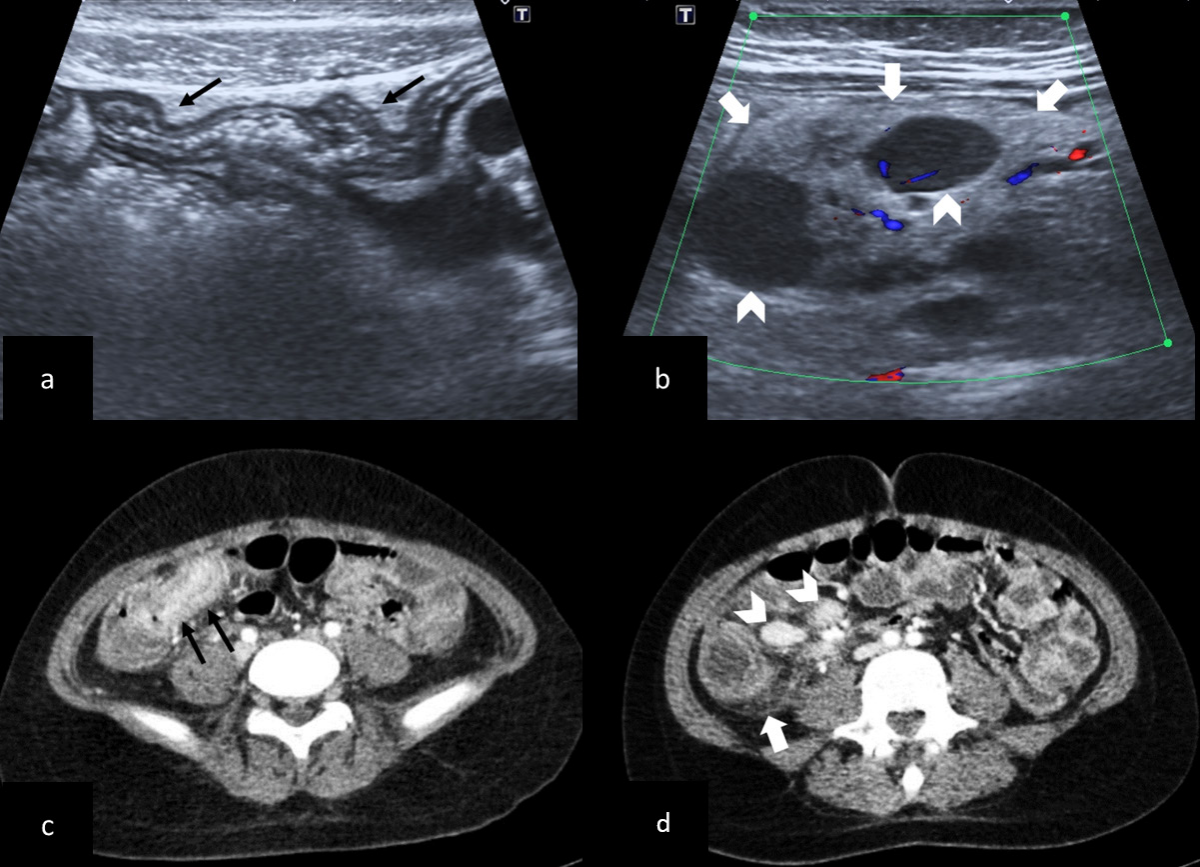
Imaging findings of an inflammation of the right iliac fossa in a 12-year-old girl who presented with fever, abdominal pain, diarrhea, and vomiting. (a–d) Ultrasonography (**a, **b) and axial CT images (**c, **d) show thickening of the terminal ileum wall (black arrows in a, c) and hyperechoic-thickened mesenteric fat (white arrows in b, d) around multiple mesenteric lymph nodes (arrow heads in b, d).

Abdominal ultrasound examinations were applied to 42 (89%) patients. The most common findings were mesenteric inflammation (thickening and increased echogenicity of mesenteric fat tissue), hepatosplenomegaly and periportal edema. In 9 (21%) patients, ileocecal wall thickening was observed, and in 7 (16%), enlarged mesenteric lymph nodes were seen. (Figure 2). In our study, the diameters of the affected lymph nodes ranged from 7 to 16 mm (median = 10 mm). Most of the lymph nodes were spherical shaped with eccentric cortical thickening and thin echogenic hilum. No abnormality was found in the Doppler echo-color study. Increased echogenicity was observed in most of the lymph nodes and adjacent soft tissue.

Hepatomegaly was observed in 16 (38%) patients. Splenomegaly and hepatosplenomegaly was observed in 12 (28%) patients.

CT examinations of the abdomen and pelvis, obtained with i.v. contrast material, were applied to 9 (19%) patients. All patients showed mesenteric inflammation. Other common findings were mesenteric lymphadenopathy and ascites.

#### Thoracic imaging findings

The thoracic imaging findings of each modality (chest X-ray, chest CT, thorax ultrasonography) are summarized in Table 3.

**Table 3.** Thoracic imaging findings with MIS-C

Thoracic imaging	Number (%)
Chest radiography	47
*Normal*	22(46)
*Pulmonary parenchymal abnormalities*	
Perihilar peribronchial thickening	22 (46)
Lower lobe atelectasis	5 (10)
Perihilar infiltration and consolidation (pneumonia)	4 (8)
*Cardiovascular abnormalities*	
Cardiomegaly	3 (6)
*Pleural abnormalities*	
Pleural effusion	4 (8)
Thorax ultrasonography	6
Pleural effusion	5 (83)
Atelectasis	3 (50)
Consolidation	3 (50)
Chest CTA	4
*Pulmonary parenchymal abnormalities*	
Normal	2 (50)
Unilateral ground glass opacification	1 (25)
Unilateral consolidation	1 (25)
Lower atelectasis	1 (25)
*Pleural abnormalities*	
Pleural effusion	1 (25)

CTA, CT angiography; MIS-C, multisystem inflammatory syndrome in children.

**Figure 3.**
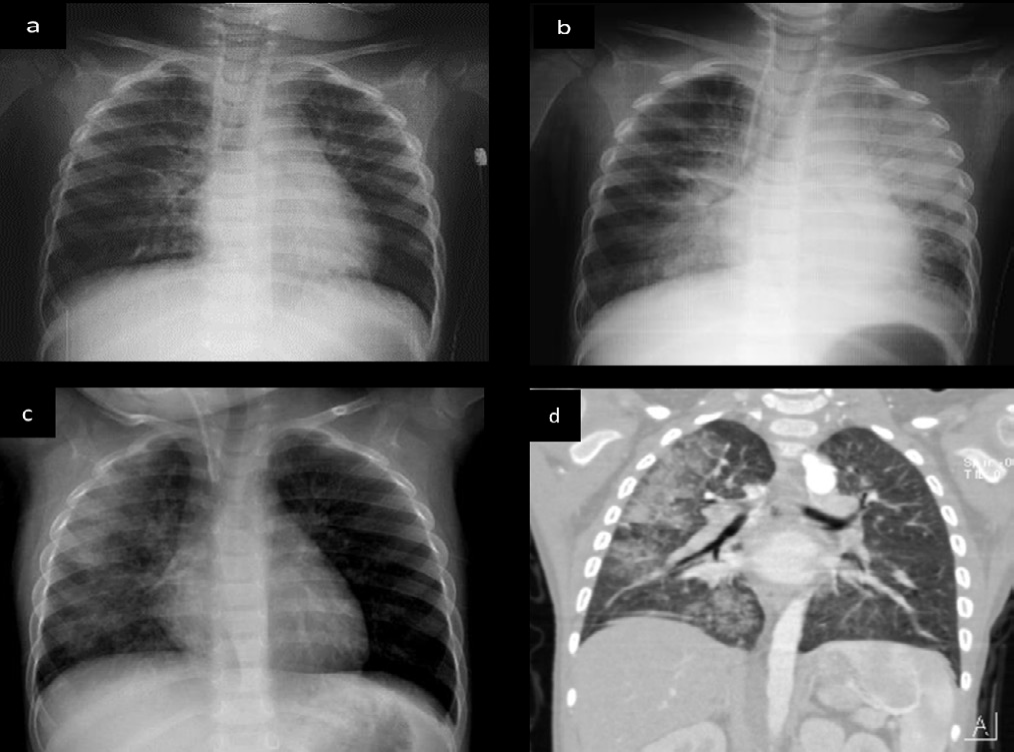
Imaging findings of a 2-year-old girl who presented with multisystem inflammatory syndrome in children associated with SARS-CoV-2, with symptoms including fever, vomiting and clinical findings of metabolic acidosis, dehydration and hypotension. (a) (initial). Frontal chest radiograph shows perihilar and paracardial peribronchial infiltration (b) (6 h later). The radiograph shows that the perihilar and paracardial peribronchial infiltration has progressed towards asymmetric, larger, consolidative opacities on the left. On radiography (c) (27 days after a and b) and tomography (d) (27 days after a and b) there was a multifocal consolidation and ground glass opacities persisting in the right lung including the cardiophrenic region.

Chest radiographs were obtained of all patients. According to the chest X-rays, 22/47 of patients (46%) showed normal findings. The most common finding was perihilar–peribronchial thickening in 22/47 patients (46%). Perihilar infiltration and consolidation were observed in 4 (8%) patients (Figure 3). Bilateral ground glass opacities and consolidation indicating acute respiratory distress syndrome (ARDS) were not seen in any patient.

Thorax ultrasonography was performed in six patients. Pleural effusion was detected in 5 (83%), in 2 (40%) of which atelectasis and collapse accompanied the effusion.

CT angiography (CTA) was applied to 4 (8%) patients. One had an elevated d-dimer level and none had pulmonary embolism. Neither mediastinal adenopathy nor hilar adenopathy was shown on chest radiography or CTA of any patient.

#### Head and neck imaging findings

The summary of the head and neck imaging findings for each modality are presented in Table 4.

**Table 4.** Head and neck imaging findings with MIS-C

Head and neck imaging	Number (%)
Neck ultrasound	4
Lymphadenopathy	4 (100)
Deep neck infection	1 (25)
Neck CT	2
Lymphadenopathy	2 (100)
Deep neck infection	1 (50)
Myositis	1 (50)
Brain CT	1
Normal	1 (100)
Brain MRI	3
Normal	2 (67)
MERS	1 (33)

MERS, mild encephalopathy with reversible splenial lesion; MIS-C, multisystem inflammatory syndrome in children.

**Figure 4.**
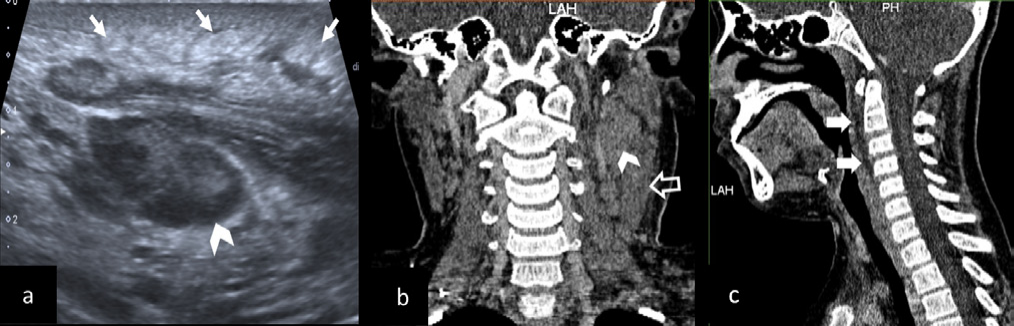
Imaging findings of a deep neck infection in a 10-year-old boy who presented with fever and swelling in the neck. (a) Ultrasonography image shows lymph nodes (arrow head) and edema of the skin-subcutaneous soft tissue planes (white arrows) (b). Coronal CT image shows lymph nodes (arrow head) and thickened sternocleidomastoid muscle (open arrow) (c). Sagittal CT image shows retropharyngeal and prevertebral edema (white arrows).

Four patients who underwent neck ultrasound had lymphadenopathy. Neck CT was performed in only one of these cases and deep neck infection was detected. Deep neck infection and myositis were seen on both ultrasound and CT in one patient. No salivary gland inflammation was observed in any of the patients (Figure 4).

**Figure 5.**
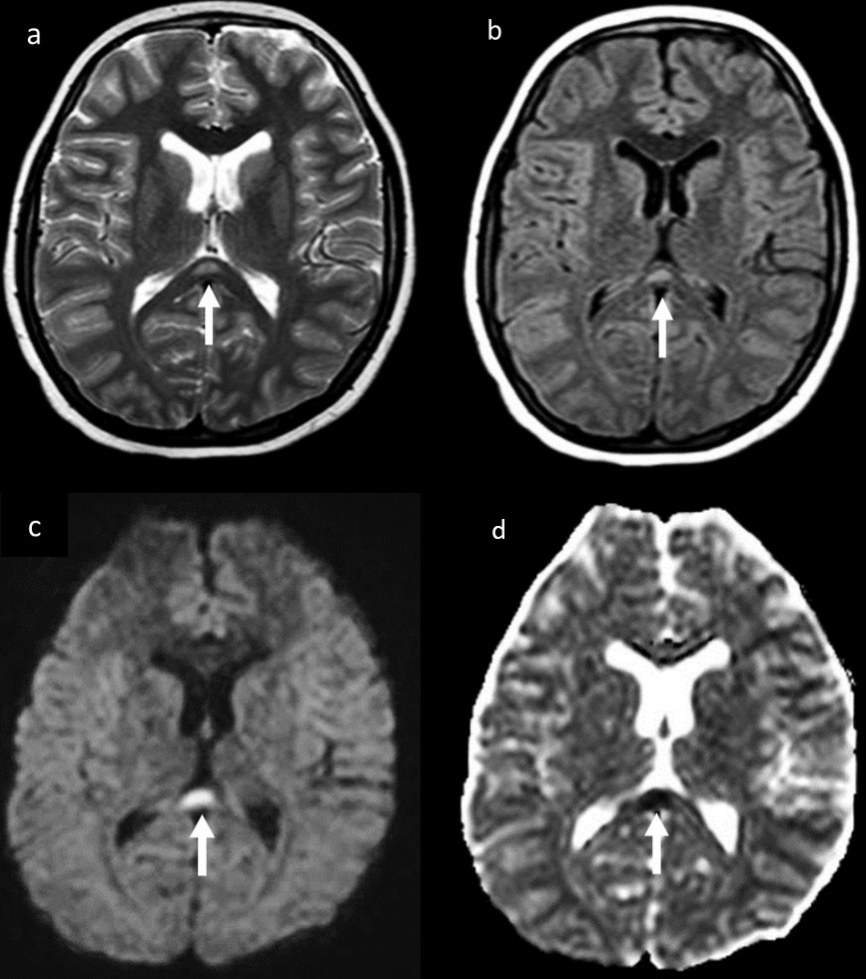
Cranial MRI of a 14-year-old boy who presented with multisystem inflammatory syndrome in children associated with SARS-CoV-2, with symptom cerebellar ataxia. A focal area of hyperintensity in the splenium of the corpus callosum on axial *T*_2_WI (a) and FLAIR (b) images corresponding to a focal area of restricted diffusion on diffusion-weighted (c) and apparent diffusion coefficient images (d). It was interpreted as MERS. FLAIR, fluid attenuated inversion recovery; MERS, mild encephalopathy with reversible splenial lesion; *T*_2_WI, *T*_2_ weighted imaging.

Brain MRI was applied to three patients. One of these patients, with cerebellar ataxia that eventually improved had restricted diffusion and T2 hyperintensity involving the corpus callosum splenium, and was diagnosed with mild encephalopathy with reversible splenial lesion (Figure 5). The lesion in the corpus callosum disappeared in the follow-up cranial MRI. Two additional brain MRIs requested for altered mental status and gait disturbance were normal.

Based on the combined results of all modalities, 22 patients (46%) had perihilar–peribronchial thickening, 18 patients (38%) had mesenteric inflammation and 16 (34%) had hepatomegaly.

## Discussion

In this study, the imaging findings of MIS-C associated with SARS-CoV-2 were evaluated in a tertiary pediatric hospital. The most common clinical findings were fever, mucocutaneous symptoms and GIT findings, respectively, which were similar to those in prior reports about MIS-C.^2–4^ In the study of Capacione et al^2^ in adults, some thrombotic events with renal and vascular findings were detected. In our study, we performed CTA with the suspicion of pulmonary embolism in a patient with respiratory distress and high INR values. However, we did not observe pulmonary embolism. In addition, we did not encounter any other renal and vascular thrombotic events. One patient presented with clinically acute epididymitis and was diagnosed with MISC. Acute epididymitis secondary to systemic diseases such as Kawasaki disease and Henoch–Schoenlein purpura has been previously reported in children.^6,7^

The majority of the patients in this study had positive serology studies indicating prior exposure and immune response to SARS-CoV-2 (*n* = 45, 95%), and two tested positive on nasopharyngeal RT-PCR (*n* = 2, 4.2%). However, none of the patients were diagnosed with acute SARS-CoV-2 infection because of the lack of pulmonary symptoms or flu-like illness. Although, two patients (4.2%) had no laboratory evidence of SARS-CoV-2 infection with negative RT-PCR and antibody results, they had a history of contact with a COVID-19 case within the 4 weeks prior to the onset of symptoms. These findings emphasize the important fact that MIS-C can present in a patient without laboratory confirmation of prior SARS-CoV-2 exposure/infection and that a positive RT-PCR result does not rule out the possibility of MIS-C, as iterated in the CDC diagnostic criteria for the syndrome.^1^

Three main thoracic imaging findings have been observed in pediatric patients with MIS-C associated with SARS-CoV-2: cardiomegaly, pulmonary edema and pleural effusions.^8^ In this study, a detailed explanation was given of extra cardiac findings, so the cardiac findings were not evaluated. In addition, no bilateral ground glass opacities and consolidation indicating ARDS were observed in any of the patients. Pleural effusion was frequently seen on all thoracic-abdominal imaging (chest radiography 4/47, thorax ultrasound 5/6, thorax CT 1/4, abdomen ultrasound 10/42, abdomen CT 3/9). In a retrospective case series by Rostad et al^9^, the radiographic features of MIS-C on the most severe chest radiographs included pleural effusions (82%), pulmonary consolidations (73%) and ground glass opacities (91%). This was thought to be because of fluid-refractory shock requiring volume resuscitation and vasoactive medications and attributable to third spacing and pulmonary edema.

Chest radiography is the most commonly used diagnostic method to evaluate lower respiratory tract infections including COVID-19 in children.^10^ All 47 children in this study underwent chest radiography at presentation due to fever and features of multisystem inflammation, and 22 of 47 radiographs (46%) were normal. On the abnormal radiographs, the most common finding was peribronchial cuffing and perihilar interstitial thickening. As none exhibited lower respiratory tract symptoms, the findings are more likely attributable to airway inflammation (22 of 47 patients, 46%). The chest radiography results closely match those of case series studies by Hameed et al^11^. In addition, in a study by Edward et al^12^, this finding was defined as the second most common finding after pulmonary opacities. It was postulated that this finding could reflect airway inflammation or pulmonary arteritis similar to that described in Kawasaki disease.

In the pediatric population, the need for chest CT for the evaluation of SARS-CoV-2 infection was not apparent, which supports the pediatric consensus recommendations of only using CT in cases where there is concern for clinical progression, an alternative diagnosis, or poor clinical improvement.^13,14^ In the current study, chest radiography was taken in all patients and a diagnosis was made from radiography. CT scans were performed in patients whose clinical condition worsened or where there was suspicion of pulmonary embolism. The hyperinflammatory state of MIS-C may create a predisposition to thromboembolic complications.^15^ Therefore, all thoracic tomography was performed with contrast to rule out the possibility of pulmonary embolism. Despite the high risk of pulmonary embolism (respiratory distress and high D-dimer) in one patient, no pulmonary embolism was detected in any of the patients.

Gastrointestinal symptoms were one of the most common features in MIS-C. Although the abdominal imaging findings are non-specific, as clinical findings overlapped with acute abdomen, abdominal imaging is performed frequently. In the current series, 42 abdominal ultrasound and 9 abdomen and pelvic CT were performed, and abdominal imaging features were described in detail. To the best of our knowledge, this is the largest series in the literature. All patients except one had abnormal findings on ultrasound and all patients showed pathological findings on CT. The most common findings were mesenteric inflammation in the right lower quadrant, hepatosplenomegaly and periportal edema on ultrasound. Ascites have been described as the most common feature in recently reported series.^1,11,12^ Interestingly, although hepatomegaly was one of the most common findings in the current study, in another large series,^12^ none of the patients demonstrated solid viscera abnormalities. However, Hameed et al^11^ reported the presence of hepatosplenomegaly and increased echogenicity of the kidneys as in the current study and unlike this study, splenic infarcts were determined. Many patients presented with multiorgan failure clinically and the presence of the hepatosplenomegaly and echogenic kidneys associated with parenchymal injury are expected findings.

Mesenteric inflammation in the right lower quadrant, and bowel wall thickening especially involving the ileocecal region are most likely due to rich lymphoid tissue and ascites were common but non-specific findings. Especially in the early cases, patients with these ultrasound findings were referred to CT for detailed examination with the suspicion of appendicitis. Nevertheless, our clinical experience increased over time and when these non-specific features were detected on ultrasound, if the clinical and serological findings met the criteria, MIS-C was included on the differential diagnosis list. Even though ultrasound is an operator-dependent modality and has some limitations especially in older and overweight patients, it can be considered sufficient for abdominal imaging in MIS-C cases.

Neck imaging was performed in a minority of the current study patients (4/47). All patients who underwent neck ultrasound had lymphadenopathy (4/47, 8.5%), which was consistent with findings in the literature.^16,17^ One patient showed an atypical initial presentation for MIS-C and presented with a deep neck infection. Although there are reports of retropharyngeal involvement in cases of MIS-C, those patients did not present with primary concerns of a deep neck infection.^18,19^ However, the current study patient had the initial diagnosis of deep neck infection without suggestion of MIS-C and subsequently met the CDC case definition for MIS-C including fever and positive SARS-CoV-2 serology.

Although many neurological symptoms associated with SARS-CoV-2 infection have been described in adults, there is little information about children.^19^ A small number of the current study patients had neurological symptoms (3/47). The symptoms of these patients were similar to those reported in the literature.^20,21^ Abdel-Abdel-Mannan et al^22^ observed transient signal changes in the corpus callosum splenium in follow-up brain MRI of 4 patients, and the same changes were observed in one of the current study patients. These findings have been reported in patients with diverse illnesses, such as Kawasaki disease and influenza, with the probable underlying mechanism being a focal intramyelin edema secondary to inflammation.

This study has some limitations, primarily the retrospective design and small sample size. In addition, although cardiovascular symptoms are the most common feature in MIS-C, the echocardiographic imaging findings were not included in the study. Furthermore, as in other studies, the features described in the study were from a localized geographic region in the world and may not represent pediatric patients in other regions, so this situation may result in differences between the case series.

## Conclusion

Although imaging findings of MIS-C are non-specific, when combined with clinical features and a history of exposure to SARS-CoV-2 associated with or without positive serological results, these imaging findings should suggest the diagnosis. Unlike typical SARS-CoV-2 infection, pulmonary manifestations are uncommon in MIS-C associated with SARS-CoV-2 and cardiovascular and gastrointestinal imaging are more important to make the correct diagnosis. In the abdominal imaging, mesenteric inflammation in the right lower quadrant, hepatosplenomegaly, periportal edema, ascites and bowel wall thickening are the most common findings. CT has been very helpful in making the differential diagnosis of the abdominal involvement of MIS-C in the early days. Radiologists should be aware of these non-specific imaging findings that may indicate the correct diagnosis of MIS-C.
